# “I Put a Lot of Emphasis on Work Because I Want to Keep My Job”: A Population‐Based Interview Study of Long Covid and Employment Changes in England

**DOI:** 10.1111/hex.70476

**Published:** 2025-10-29

**Authors:** Viveka Guzmán, Chiara Di Gravio, Emily Cooper, Adam Lound, Nikki Smith, Margaret O'Hara, Christina J. Atchison, Graham Cooke, Marc Chadeau, Paul Elliott, Helen Ward

**Affiliations:** ^1^ Department of Epidemiology and Biostatistics, School of Public Health Imperial College London London UK; ^2^ Patient Experience Research Centre, School of Public Health Imperial College London London UK; ^3^ National Institute for Health Research Imperial Biomedical Research Centre London UK; ^4^ REACT‐LC Public Advisory Group, School of Public Health Imperial College London London UK; ^5^ Department of Infectious Disease Imperial College London London UK; ^6^ MRC Centre for Environment and Health Imperial College London London UK

**Keywords:** employment, England, health inequalities, lived experiences, long Covid, REACT study

## Abstract

**Background:**

Long Covid is a complex condition characterised by persistent multisystemic symptoms following a Covid‐19 infection, which can influence an individual's capability to sustain employment. However, there is limited evidence of how diverse presentations of long Covid can shape employment and what support strategies might be useful for different groups.

**Aim:**

To address this, we aimed to explore the experiences of employment changes among people living with long Covid in England and to identify the perceived barriers and enablers they face to cope with work.

**Design and Methods:**

We conducted a qualitative analysis of data from the Real‐time Assessment of Community Transmission (REACT) Study. Using a framework analysis approach, we analysed 60 semi‐structured interviews with people who experienced persistent Covid‐19 symptoms for 12 weeks or more.

**Results:**

We identified three key themes: (1) Persistent Covid‐19 symptoms at work; (2) Ripple effects of balancing work, identity and well‐being with persistent Covid‐19 symptoms; and (3) Employment changes to cope with and manage persistent Covid‐19 symptoms. Participants identified multiple employment changes, including reduction of working hours, restructuring of roles and modification of responsibilities, and adapted ways of working. Drivers of employment changes included disruptive and fluctuating symptoms but also broader pandemic circumstances and the opportunities available for accessing organizational support and putting in place appropriate management strategies.

**Conclusion:**

Our results provide a thorough understanding of the work changes experienced by people living with long Covid and highlight the need for intersectional, adaptable work accommodations to support their sustainable employment and overall well‐being.

**Patient and Public Contribution:**

Members of the public who are part of a Public Advisory Group (PAG) have provided ongoing input into various aspects of the umbrella cohort study, the Real‐time Assessment of Community Transmission (REACT) Study, including the study design, data collection instruments and dissemination of findings. For this qualitative study, which draws on interview data from REACT Long COVID (REACT‐LC), preliminary findings were presented to the PAG for feedback and suggestions, which helped refine the discussion. Additionally, two Public Advisors with lived experience of long Covid contributed to the writing and editing of this manuscript. In accordance with these contributions, they are included as authors.

## 1 Background

For millions of people worldwide, SARS‐CoV‐2 infection has led to a variety of chronic, systemic and often disabling symptoms, collectively known as long Covid [1]. The 2024 National Academies of Science, Engineering, and Medicine (NASEM), building on an earlier definition by the World Health Organisation [2], defines long Covid as ‘an infection‐associated chronic condition that occurs after SARS‐CoV‐2 infection and is present for at least 3 months as a continuous, relapsing‐remitting, or progressive disease state that affects one or more organ systems’ [1]. The risk of persistent symptoms following an episode of Covid‐19 has been estimated to be around 6% [3], with 400 million people affected worldwide by 2024 [4]. In the United Kingdom, 4.8% of nearly 760,000 surveyed participants aged 16 and over reported long Covid in 2024, while an additional 9.1% experienced persistent Covid‐19 symptoms but were unsure of their diagnosis [5]. Individuals living with long Covid may face substantial limitations in their daily activities, with one UK study reporting that 31.7% experienced severe limitations, compared to 12.2% of those without Covid [6].

Long Covid has also been shown to have a substantial impact on employment, as symptoms like fatigue, cognitive dysfunction and post‐exertional malaise can preclude individuals from working or limit the amount and type of work they can undertake [7, 8, 9]. The European Commission estimated that in 2022, long Covid reduced the EU labour supply by 0.3%–0.5%, leading to a 0.2%–0.3% decline in economic output [10]. Similarly, evidence from the United Kingdom suggests that since the start of the pandemic around 80,000 people have left employment for causes directly attributable to long Covid [11]. Previous evidence suggests that reduced working hours among people with long Covid in the United Kingdom have resulted in a mean income decline of 24.5%, equivalent to £5.7 billion in national losses [12].

Recognition of the devastating financial, personal and social consequences of long Covid has prompted attention to employees lived experiences, particularly the barriers and facilitators they face to return to work [13, 14, 15]. This body of work suggests that individuals strive to remain in employment despite work‐disrupting symptoms, not only due to financial need but also the desire to maintain a sense of purpose and their personal or group identity [13, 14]. Barriers identified to sustaining employment with long Covid include disbelief and stigma about the condition, difficulties in accessing diagnostic tests for acute Covid‐19 (which can hinder recognition of persistent symptoms as long Covid), limited services and supportive networks at the organizational level, and lack of appropriate legislative and policy protections, such as anti‐discrimination laws, sick leave and disability benefits, and flexible work regulations [15, 16].

Although existing evidence highlights promising strategies to support individuals facing challenges to return‐to‐work due to ongoing long Covid, there is limited guidance for supporting workers undergoing other employment changes. These include employees experiencing severe or chronic disabilities that impair their capacity to perform job functions, or those transitioning to medical or early retirement. Additionally, most previous studies recruited participants from long Covid support groups or clinical services. Thus, the experiences of workers managing their symptoms outside formal support remain underexplored. Addressing these knowledge gaps will contribute to identify interventions that provide adequate support for workers facing health‐related employment changes, enabling their sustainable employment or transition to alternative pathways when needed.

In this paper, we use population‐based interview data from the REACT Long COVID (REACT‐LC) Study [6, 17] to provide insights related to the experiences of employment among people in England with persistent Covid‐19 symptoms, consistent with established definitions of long Covid [1, 2]. The specific objectives of this analysis are to explore the influence of long Covid on individual's employment changes and to identify the perceived barriers and enablers related to their symptom management and capability to sustain employment.

## 2 Materials and Methods

### 2.1 Study Design

**Figure 1:**
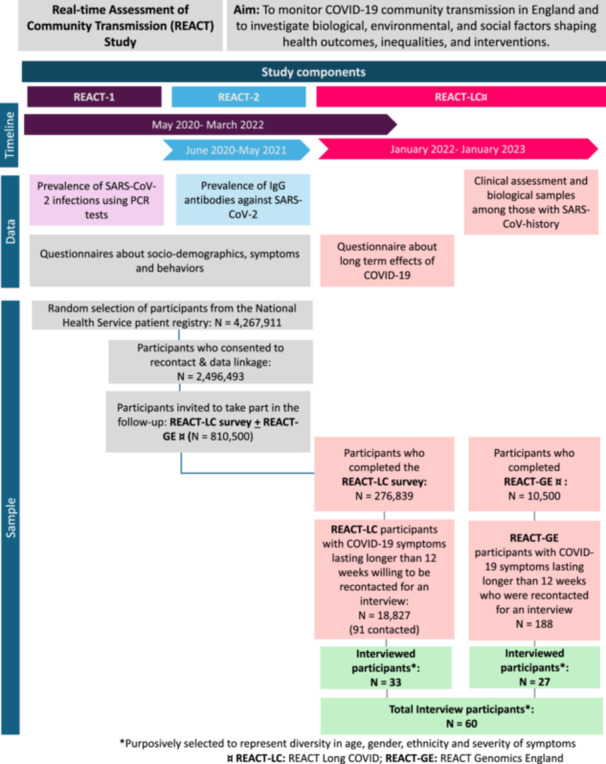
Overview of the REACT Study and flow chart from the broader cohort to the interview study sample (*N* = 60). *Purposively selected to represent diversity in age, gender, ethnicity and severity of symptoms. **REACT‐LC:** REACT Long COVID; **REACT‐GE:** REACT Genomics England.

This study is a secondary analysis of pre‐existing interview data collected within the REACT Study, a broader population‐based cohort described elsewhere [18, 19, 20]. Figure 1 presents an overview of REACT components and the process by which the final sample was derived (*N* = 60). A comprehensive account of the interview study methodology has been reported previously [21]. Briefly, interview participants were drawn from the REACT‐LC sample based on eligibility criteria. To be considered, individuals must have experienced persistent symptoms lasting 12 weeks or more following symptomatic Covid‐19 (regardless of diagnosis status), attended an assessment clinic and provided biological samples and/or completed a Health and Wellbeing survey, and consented to be recontacted for an interview. To capture a wide range of experiences, the sampling approach purposively selected individuals to reflect diversity in age, gender, ethnicity and symptom severity. For the present analysis, no additional sampling was conducted, and data saturation did not guide participant selection. This approach aligns with established guidance for secondary analyses, where the analytic focus is on re‐examining existing data rather than generating new data [22, 23].

Interviews used a semi‐structured topic guide that was developed in line with previous literature, a pilot interview study [24], and feedback from the REACT Public Advisory Group (PAG). It comprised questions related to participants' history of Covid‐19, pre‐pandemic and pandemic life experiences, persistent Covid‐19 symptoms, perceived trajectories of these and management strategies [25]. Interviews were conducted online either via MS Teams or Zoom, between January 2022 and January 2023. Each interview lasted around an hour and was recorded for professional transcription.

### 2.2 Analysis

We selected framework analysis as our analytical approach due to its suitability to manage a relatively large dataset and its allowance to incorporate both a priori and emerging codes [26]. NVivo 14 software [26] was used to organise the data, assist coding and document our analytic decisions. We followed the five stages of framework analysis outlined by Ritchie and Spencer [27]: (1) Familiarised with all transcripts and field notes; (2) Developed initial codes using a combined deductive‐inductive approach shaped by our research question and prior literature, while allowing new codes to emerge [28]; (3) Iteratively refined our codebook by consolidating codes into broader thematic categories and then applied it to the remaining transcripts; (4) Developed a framework matrix to summarise each participant's employment experiences; and (5) Finalised themes by identifying patterns within and across participants.

Coding was primarily conducted independently by the lead author, with co‐authors providing input on the final themes. Interrater reliability was not formally assessed; instead, rigour was supported through regular presentation of preliminary analyses and interpretations to the wider research team for discussion and critical feedback [28]. Illustrative quotes were selected based on representativeness of patterns and themes, clarity of expression, and diversity of perspectives, including negative or minority cases [29, 30, 31]. The lead author carried out the initial quote selection, with feedback from PAG co‐authors prompting some alternative choices to ensure better alignment with the criteria.

We reflected on our positionality as researchers and the potential impact of our own experiences, assumptions and biases in the data collection, analysis and interpretation. Individual reflections and group discussions were recorded in our research log and allowed us to consider our insider/outsider perspectives, track our analytic decisions and iteratively adapt our methods.

## 3 Results

The 60 people who were interviewed included 37 women and 23 men (including 1 transgender man), aged between 18 and 80 years (median 46). At the time of the interview, 48 participants were working (26 full‐time, 13 part‐time and 9 self‐employed) across diverse sectors, with 25 in non‐public facing roles. Table 1 presents additional socio‐demographic and health characteristics [32].

**Table 1:** Summary of participants socio‐demographic and health characteristics.

	*N* (%) or Median (IQR)
Employment nature at recruitment in REACT	
Full‐time	26 (43%)
Part‐time	13 (22%)
Self‐employed	9 (15%)
Unemployed, studying or retired	12 (20%)
Employment type at recruitment in REACT**	
Service, logistics and security	3 (5%)
Child‐related	4 (7%)
Healthcare worker	6 (10%)
Other essential worker/public facing	9 (15%)
Work at home/not public facing	25 (41%)
Not in full‐time, part‐time nor self‐employed	12 (20%)
Gender	
Male	23 (38%)
Female	37 (62%)
Age (years)	46 (33, 56)
Ethnicity	
Asian	9 (15%)
Black	8 (13%)
Mixed	12 (20%)
Other	6 (10%)
White	25 (42%)
IMD Quintile	
Q1—most deprived	7 (13%)
Q2	10 (18%)
Q3	13 (24%)
Q4	12 (22%)
Q5—least deprived	13 (24%)
Missing	5
Covid‐19 infection severity	
Mild‐moderate symptoms	26 (43%)
Severe symptoms	34 (57%)
Number of comorbidities	
0–1	40 (50%)
2+	17 (30%)
Missing	3
Symptom duration since acute Covid‐19 infection¤	
Among participants who identify as recovered (*n* = 7)	
≥ 12 weeks to < 1 year (52 weeks)	4 (7%)
≥ 1 year < 2 years	3 (5%)
Among participants with ongoing symptoms (*n* = 53)	
≥ 12 weeks to < 1 year	8 (13%)
≥ 1 year < 2 years	24 (40%)
≥ 2 years	21 (35%)

* Except for symptom duration since acute COVID‐19 infection^**¤**^, participant's characteristics were summarized from their answers to the REACT‐LC questionnaire, except for three participants who did not complete the questionnaire and for whom we considered their interview responses.

^¤^ Extracted from interview responses.

** Business and service includes individuals working in food retail, hospitality and personal care. Child‐related includes individuals working in schools, nurseries, childcare centers or providing childcare services. Logistic and security includes individuals working in home deliveries, public transit, police, prison, fire and rescue, coastguard, and armed forces. Healthcare workers and care home workers include both workers with and without contact with the public. Other essential workers/public facing roles include all other essential workers that were not included in the other groups based on government guidelines (32).

**Figure 2:**
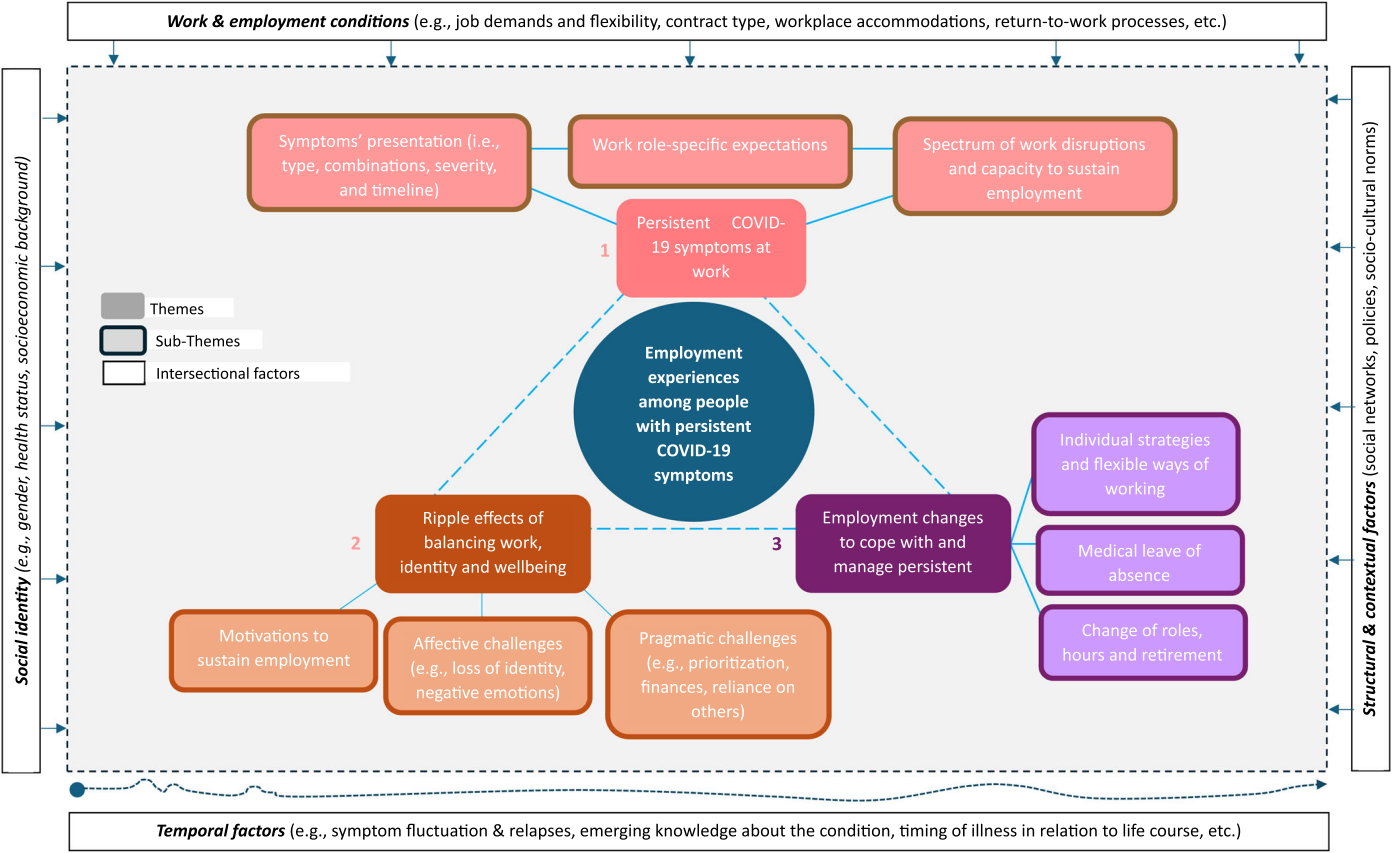
Thematic map of participants' employment experiences with persistent Covid‐19 symptoms.

Participants described diverse employment changes since the start of the Covid‐19 pandemic and recognized their persistent symptoms as important drivers of these. Figure 2 shows the thematic map of our analysis, which identified three overarching themes: (1) Persistent Covid‐19 symptoms at work; (2) Ripple effects of balancing work, identity and wellbeing with persistent Covid‐19 symptoms; and (3) Employment changes to cope with and manage persistent Covid‐19 symptoms. Themes are described below and illustrated with quotes from participants. Quotes are presented with pseudonyms, age range at the time of data collection, and employment nature before the pandemic (FT = full‐time employment; PT = part‐time employment).

### 3.1 Persistent Covid Symptoms at Work

This theme captures participants' perspectives about how their persistent Covid‐19 symptoms intersected with their ability to meet work expectations. The symptoms most commonly described as disrupting work were physical fatigue, diminished cognitive capacities (e.g., brain fog, poor memory and mental fatigue), breathlessness and pain. Symptoms manifested in diverse combinations, severities and timelines, shaping participants' capabilities to carry out work to varying degrees. Some participants reported they felt unable to deliver outputs to their previous standard, as Vivian (46–55 years, FT) illustrated: ‘I'm not functioning at the same levels as I used to. Stuff that I used to knock off in a couple of hours is now taking me weeks’. A sense of diminished capacity to meet work demands was echoed by others, not only in terms of time taken to complete tasks but also in the need to repeatedly revisit and relearn material long familiar to them. Elaine (46–55 years, FT), who worked as an exhibition's curator, described:

I've done my job for 30 years and I'm very competent at it, but I couldn't understand what some of the words were meaning. I had to reread about stuff. I was thinking I'd forgotten about things. It was more than just being out of touch […] It's almost having to recognise things again and relearn things which I struggled with a lot.

Others reported that the severity of their symptoms, combined with the demands of their roles, made it impossible altogether to sustain their previous employment. Jasleen (56–65 years, FT), who worked teaching vulnerable people, illustrated: ‘I couldn't entertain work now, at all, because it would just be‐ especially the kind of work I do. The work I do, it gives out a lot of energy’. Several other participants also used ‘energy’ to describe finite functional reserves necessary to meet work demands, which were often depleted by their symptoms.

Beyond cognitively demanding roles, work disruptions were also pronounced among participants employed in public‐facing positions, where physical demands, immediacy of response and greater reinfection risks exacerbated challenges to continue working. This was often reported among healthcare workers and those in retail or service positions. Vinay (46–55 years, FT), who worked in a hospital, reflected on this, noting that ‘it was frightening because being on a ward is not something that you could just think of not doing, moving around and being active’.

Additionally, participants described work disruptions related to fluctuations of their symptoms through time, non‐linear recovery trajectories, and uncertainty on the overall course of the condition. Some of the participants also perceived that work had sometimes caused a relapse or exacerbation of their symptoms, which was often associated with a quick return to work or ‘overdoing it’. For instance, Tasha (46–55 years, FT) who held a position within a charity organization, described each time she would try to return to work as ‘things were getting better’, her symptoms would ‘go backwards’ and she had to take additional time off, which affected her emotionally and financially:

I was trying to work. So, I would work and I'd be off, I'd work and I'd be off, and I'd just be going through this yoyo existence. I'd manage a couple of months of work and then I'd have to be off again for a month […] so income was still really sporadic.

### 3.2 Ripple Effects of Balancing Work, Identity and Well‐Being With Persistent Covid Symptoms

This theme details the consequences of participants' ‘struggles to get through’ work, including the emotional, physical and social tolls involved in balancing job demands with persistent symptoms. Some participants reported losing self‐confidence in their abilities to perform previous work tasks, which led to feelings of frustration, low mood and anxiety. As Dolores (36–45 years, PT) illustrated: ‘I'm a bit of a person who doesn't want to be seen as not doing something correctly, I like to do a job to the best of my ability. And feeling that you're not necessarily doing that to the best of your ability is frustrating’. Similarly, participants reported facing or fearing an increase in their work mistakes, so some verified their outputs multiple times which often led to additional stress and more time needed to complete a task. Sabina (46–55 years, FT), who's work involved dealing with numbers, explained:

I could not concentrate, I had to keep on doing things over and over again, just to make sure that I was doing it right. Which kind of stressed you out, because you think, “Oh my God, I've got such a responsibility here”.

Changes in participants' capabilities challenged their established roles within their work teams. Colleen (36–45 years, FT), a project manager operating at the interface of environmental policy and science, remarked:

It was noticeable to my peers as well that I wasn't quite as sharp as I usually am. You know, I'm the one that never says anything inappropriate at a meeting, but it wasn't that I was inappropriate. I'm the people person. I'm the communicator. I'm the one that will explain this policy, you know, in simple terms, and I just … yes, it was difficult. It was hard work.

In the face of these challenges, several participants felt that leaving their employment was not an option for them, mostly due to a combination of financial circumstances, and wishing to maintain their professional identity and sense of purpose. This resulted in participants attempting to reconcile work expectations with their symptoms and daily life, which required them to plan, prioritize and iteratively appraise their activities. Danielle (46–55 years, FT) explained:

I put a lot of emphasis on work because I want to keep my job, and I want to be able to at least have some income, but work means […] there's no energy left, at the end of the day, to do that [*laundry and other home chores*].

Limited energy and prioritization of work reduced participants' opportunities to engage in other valued activities, such as recreation and leisure, which previous research suggests may contribute to long‐term decline in physical and mental well‐being [33] and in work ability [34]. This also increased reliance on social networks for instrumental support with home and caregiving tasks, potentially generating additional challenges for women and migrant individuals, who are more often expected to assume primary responsibility for these roles [35, 36].

Taken together, challenges in balancing employment with fluctuating symptoms and non‐linear trajectories placed participants, such as Elaine (46–55 years, FT), in a state of uncertainty regarding their identity and career prospects:

What are we now, 6 months into returning and I'm still only doing 2 days a week. I wondered will I ever get back up? Hopefully I can get back up to things, but I don't think I'll probably return to be, maybe I will, I don't know, the person I was before.

### 3.3 Employment Changes to Cope and Manage Persistent Symptoms

This theme describes participants' perspectives of individual and organizational strategies for managing persistent symptoms at work, as well as perceived barriers or enablers influencing their implementation.

#### 3.3.1 Individual Strategies and Flexible Ways of Working

Participants used diverse strategies to sustain employment, including adaptive approaches that helped them manage challenges constructively, and maladaptive strategies that provided short‐term relief but ultimately lead to negative outcomes [37, 38]. Examples of maladaptive practices included ‘pushing through’ symptoms, avoiding communication about health needs, and working longer hours to compensate for reduced efficiency. Liam (46–55 years, FT) explained: ‘What I was doing was just working harder and later and trying to hide the fact of what was going on’. In contrast, other participants, including Colleen (36–45 years, FT), sought to manage expectations and communicated their need for accommodations to employers and colleagues: ‘I allowed myself a bit more time where possible, and generally just prefaced meetings with, “Sorry, I've got COVID brain. Bear with me whilst I figure out what I'm trying to say”’. Consistent with prior research on workplace disclosures [39, 40], participants noted disclosure was complex and involved weighing potential risks (e.g., stigma and career disadvantages) against benefits (e.g., accommodations and support).

Participants also reported that awareness of factors influencing their symptoms developed over time, prompting them to tinker with their management strategies. Commonly reported enablers included scheduled breaks and pacing activities over multiple days. Rita (46–55 years, FT), whose role involved occasional travel, illustrated:

If I put a couple of things back‐to‐back, I'm knocked out for a couple of days. So, my boss, again super‐understanding, is sparing about when they use me for a meeting in London. It doesn't tend to be an internal thing, it's usually for something external‐facing where I can be a value‐add.

Online and remote working conditions often provided improved opportunities for sustaining employment. Yet, participants also described limitations, such as screen fatigue, which prompted compensatory strategies. Naomi (46–55 years, FT), for instance, shared she found Zoom meetings exhausting, ‘especially if there are lots of people’ and indicated that rather than attending these, she kept track of team developments through email and meeting minutes. Other barriers for remote working included lack of physical resources, such as reliable electronic devices or a stable internet connection, which Jim (46–55 years, FT) illustrated:

I was quite happy to work from home, if you like, so I wasn't on the sick but I'd be working from home. They couldn't find me anything, they couldn't get me a laptop out […] In the end, I just had to stay on the sick because I couldn't come in. It's a shame really because I was quite happy to go back to work.

#### 3.3.2 Medical Leave of Absence

For several participants stopping work for some time, ranging from a few days to months, was necessary to focus on their recovery. David (46–55 years, FT), a sales professional, illustrated:

There was just no way I could carry on. When it happened and I rang my boss and said, “I now need to go sick, I can't see the wood for the trees at the moment.” It was a massive relief actually, it was really quite an enlightening thing to do once you've made the decision that you can't do it.

However, access to medical leave varied, with self‐employed and precariously employed participants facing structural barriers previously documented in the literature [41], which Joy (26–35 years, FT) illustrated: ‘The company I work for don't offer any sick pay, and I was more worried about earning money and paying my bills than taking the proper time off, to look after myself’. Additionally, participants reported that deciding to take leave often required a consultation with an occupational therapist or physician, which were often contingent on access to these services and receiving a long Covid diagnosis. This highlights how systemic structures may intersect with health to create precarious contexts [21, 42].

Participants frequently reported that taking medical leave was accompanied by feelings of guilt over burdening already strained co‐workers with additional duties, concerns about career impact, and navigating cultural or organizational barriers to discussing their symptoms. Sarah (26–35 years, FT), who worked as a teacher, explained:

I didn't feel comfortable, to be honest, to phone school and say that I'm too tired to teach, even though, generally, this was a physical symptom […] Again, probably having that Long COVID diagnosis would allow you to do that. Right now, it's very difficult to phone school and say, “I'm too tired to work”.

Similarly, Debbie (56–65 years, FT), a Health Service employee described how the prevailing culture at her workplace—‘You've just got to brush yourself off and get on with things’—pressured her to return to full‐time before she felt ready. These accounts illustrate how pervasive social stigma against invisible diseases and a lack of recognition of long Covid as a potentially long‐term, disabling condition hindered access to appropriate support. Moreover, participants such as Clara (36–45 years, FT) highlighted these challenges created a looping effect which contributed to further health issues and employment disruptions:

I cannot remember a time since COVID where I didn't have either a migraine or a really bad headache every single week. It just got so bad where I was starting to get palpitations and other cardiac symptoms that I just decided it was enough. I mentioned it to my manager, and they then said that the COVID policy had changed, and if you went off with COVID, it was just normal illness that could trigger sickness that would then affect your job.

In contrast, other participants, such as Eunice (56–65 years, FT) reported their ‘employers were quite understanding’ and allowed them to rebuild working stamina and confidence gradually. However, some participants perceived that although their managers and co‐workers seemed keen to support them, they had limited guidance and knowledge about the best strategies to do so.

#### 3.3.3 Change of Roles, Hours and Retirement

Several participants reported that due to their persistent Covid‐19 symptoms they had made job transitions or changed their hours of work, including internal transfers within the same organization, switching employers, and retiring. Some participants considered their symptoms as the main driver of these changes, while others viewed them as the tipping point that accelerated circumstances already set in that direction. For instance, Clara (36–45 years, FT), who moved from a job in a hospital to a new position based in a community setting, said:

The main driver for me leaving was because of my COVID symptoms and how I wasn't able to properly manage it in my old role, to a point, like I said, [*I took*] a substantial pay cut, just to try and balance things off. Because I knew it would have taken an awful toll on my long‐term health if I wasn't to change something, and I knew it wouldn't happen in the hospital.

Since starting her new job in a community setting, Clara's working circumstances had dramatically improved:

In the short time I've worked there, they've been a lot more sympathetic, without me even having to explain what I'm going through. They've been very accommodating with regard to how to manage my caseload, how to adjust my work hours to accommodate times when I may not necessarily be at my best, and how to ensure that I cover everything I need to.

Similarly, other participants reported a prioritization of their physical health over employment continuation although this sometimes led to mixed feelings. For instance, James (46–55 years, FT) who went into early retirement, shared: ‘It was disappointing I couldn't go in and finish my career in the right way but, at the same time, what I did think about was, “I've done 29 years for you, I'm going to look after me”’. Decisions to retire or diminish working hours were often balanced with regard to personal finances, social networks (e.g., dependents and life partners) and medium‐ to long‐term life plans. Participants, such as Laila (56–65, F, FT), also highlighted the value of having the opportunity to decide how or when to reduce their working hours:

Because I had a choice, I was able to negotiate retiring, but I said, “But I still want to work and I want to work 2 days a week,” so they gave me a 2‐days‐a‐week contract.

Similarly, other participants reported that having agency over their roles and working hours allowed them to continue to benefit from employment ‘in their own terms’, which some perceived as an opportunity to ‘take their mind off’ their symptoms (Jim, 46–55 years, FT) or to ease into more permanent transitions. Others recognized that indefinite leave was the more appropriate decision and that their employers could help them to ‘leave work well’. Tom (56–65 years, FT) began considering retirement following critical disruptions to his work capabilities due to severe cognitive symptoms and tiredness, with little improvement despite a period of medical leave and phased return to work. In response, his manager supported him with personalized follow‐ups and space to consider his options:

He didn't believe I should be making those types of decisions feeling the way that I did, so he encouraged me to stay. I think September came along and I rang him up again and said, “No, I'm definitely not coming back, I need to stop.” He said, “I'll help you with that.” […] I'd been in the company for 23 years. He managed to get me a golden handshake so December 31st of 2021 I left. I'm not working now […] And things have definitely picked up since then.

## 4 Discussion

This study provides insights into employment changes experienced by people living with persistent Covid‐19 symptoms, highlighting enablers and barriers they face in managing symptoms at work. Heterogeneous symptom presentations meant that some participants were completely unable to sustain employment, while others faced increased work instability as fluctuating symptoms misaligned with job demands. Beyond this interplay, employment changes were shaped by access to formal supports and opportunities to leverage social capital and informal resources. Interactions between these factors influenced the coping strategies participants deployed, while simultaneously creating additional burdens or respite opportunities as they navigated symptom management and sought support. The study further emphasizes how work environments shape the employment trajectories of people with persistent Covid‐19 symptoms, particularly in public‐facing roles and sectors such as healthcare and education, which are associated with intense workloads, understaffing and higher Covid‐19 exposure [43, 44, 45]. Precarious working conditions can compound these challenges, especially when support systems are lacking [46]. This is consistent with previous evidence, indicating that self‐employed individuals were less able to take days off to recover due to limited social benefits and concerns about losing their clients or income [15].

By highlighting the challenges faced by workers with persistent Covid‐19 symptoms, our findings contribute to the broader evidence‐base on chronic diseases, disability and employment [47, 48]. Specifically, findings underscore the importance of flexible work accommodations and the official recognition of potentially disabling effects [49]. These needs overlap with those from other health conditions characterised by fluctuating symptoms and stigmatisation, such as chronic fatigue syndrome and fibromyalgia [50, 51]. These conditions require distinct approaches from other chronic illnesses due to their episodic, multisystemic nature which may produce unpredictable challenges to sustain employment [50, 52]. Thus, resources for iteratively tailoring accommodations and flexible management strategies should be readily accessible to employers and occupational health professionals, enabling them to support affected individuals effectively and to provide guidance for their social and professional networks [49]. However, access to organizational supports often depends on formal recognition of disability, which can place additional burdens on affected individuals [52].

In the United Kingdom, recognition of long Covid as a potential disability under the Equality Act represents a crucial step towards enabling such support. Yet, the case‐by‐case approach requires individuals to provide medical evidence and disclose their condition to employers [53]. Findings from our study indicate that this additional burden, on top of managing symptoms, may exacerbate health problems and workplace challenges. Nonetheless, participants' accounts suggest that proactive workplace measures, including dynamic workload planning, regular check‐ins and efforts to reduce stigma, can bridge the gap between legal recognition of disability and meaningful, day‐to‐day support, enabling individuals to sustain employment while managing health challenges.

Based on these results, we suggest an intersectional approach is needed to identify workers with long Covid who might face unique challenges. This approach should consider potential interactions between relational and contextual characteristics to develop work accommodations and tailored supports based on iterative appraisals of individual's needs [54]. Interventions should expand beyond return‐to‐work support since many individuals continue to experience fluctuating symptoms and challenges to manage their symptoms, which can also interfere with life–work balance and long‐term well‐being [49, 54]. According to participants' perspectives, useful strategies include flexible ways of working, such as task scheduling responsive to day‐to‐day symptoms, multi‐day pacing of activities, hybrid working opportunities, and agency in setting productivity expectations. These strategies should be part of a broader toolkit of workplace accommodations [49] that should be accompanied by wider employment policies and access to holistic support services, including within the NHS [55].

### 4.1 Strengths and Limitations

The purposive sampling approach used to recruit participants to the REACT interview study enabled us to explore a wide variety of employment experiences among people with persistent Covid‐19 symptoms, including individuals with heterogeneous demographic backgrounds, diverse employment types, and varying access to long Covid support groups and clinical services.

The cross‐sectional study design of our study provides limited scope to explore the long‐term effect of long Covid in employment. This should be addressed in future studies as the condition is characterized by potential relapses and knowledge about the condition continues to emerge in the face of changing socio‐economic conditions that may affect opportunities for sustainable employment. Although our sampling approach allowed us to recruit participants from several regions of England and yielded a substantial sample by qualitative standards [56, 57], the purposive and self‐selective nature of recruitment may have introduced selection bias and constrained representativeness [58]. As such, our findings should be considered with caution and triangulated with longitudinal evidence and studies from different political and economic contexts. Moreover, we consider that understanding the employment experiences of people with long Covid also requires the consideration of the perspectives from other relevant stakeholders not included in this analysis, such as carers and family members, line managers, co‐workers, occupational therapists and clinicians.

## 5 Conclusion

Findings from our qualitative analysis provide an in‐depth characterization of people's experiences navigating their work alongside persistent Covid‐19 symptoms, as well as the barriers and enablers they have experienced to put in place adaptive strategies. These results offer valuable insights for developing workplace accommodations that support sustainable employment and well‐being of working‐aged adults living with long Covid. We propose that an intersectional approach, which considers symptom presentation, job responsibilities, healthcare access and social networks, is essential for developing effective, tailored work support and policies for persistent Covid‐19 symptoms. Given the changing landscape related to long Covid, it is crucial that workplace support and policies remain adaptable to meet the evolving needs of workers with persistent symptoms and that they collaborate with those with lived experience in their development and implementation.

## Author Contributions

Conceptualization: V.G., C.D.G. and H.W. Methodology: V.G. Formal analysis: V.G. Investigation: V.G. and C.D.G. Resources: E.C. and A.L. Data curation: V.G., E.C. and A.L. Writing – original draft: V.G., C.D.G., N.S., M.O'.H. and H.W. Writing – review and editing: V.G., C.D.G., E.C., A.L., N.S., M.O'.H., C.J.A., G.C., M.C., P.E. and H.W. Project administration: V.G. and C.D.G. Funding acquisition: G.C., M.C., P.E. and H.W.

## Disclosure

The views expressed in this publication are those of the authors and not necessarily those of DHSC, NIHR or UKRI.

## Ethics Statement

The REACT and REACT‐LC Studies hold ethical approval from South‐Central Berkshire B Research Ethics Committee (IRAS IDs: 298404, 259978, 283787 and 298724).

## Consent

All participants provided informed consent to take part in the study.

## Conflicts of Interest

The authors declare no conflicts of interest.

## Data Availability

The data that support the findings of this study are not publicly available. De‐identified data can be made available upon reasonable request and in line with the consent agreed with participants, by submitting a methodologically sound research proposal to react.lc.study@imperial.ac.uk.
